# Stem Cell Applications in Tendon Disorders: A Clinical Perspective

**DOI:** 10.1155/2012/637836

**Published:** 2012-01-29

**Authors:** Mark Young

**Affiliations:** ^1^Department of Biotherapies, Mater Medical Research Institute, Aubigny Place, Raymond Terrace, South Brisbane, QLD 4101, Australia; ^2^Qsportsmedicine, GPO Box 96, Brisbane, QLD 4000, Australia

## Abstract

Tendon injuries are a common cause of morbidity and a significant health burden on society. Tendons are structural tissues connecting muscle to bone and are prone to tearing and tendinopathy, an overuse or degenerative condition that is characterized by failed healing and cellular depletion. Current treatments, for tendon tear are conservative, surgical repair or surgical scaffold reconstruction. Tendinopathy is treated by exercises, injection therapies, shock wave treatments or surgical tendon debridement. However, tendons usually heal with fibrosis and scar tissue, which has suboptimal tensile strength and is prone to reinjury, resulting in lifestyle changes with activity restriction. Preclinical studies show that cell therapies have the potential to regenerate rather than repair tendon tissue, a process termed tenogenesis. A number of different cell lines, with varying degrees of differentiation, have being evaluated including stem cells, tendon derived cells and dermal fibroblasts. Even though cellular therapies offer some potential in treating tendon disorders, there have been few published clinical trials to determine the ideal cell source, the number of cells to administer, or the optimal bioscaffold for clinical use.

## 1. Tendon Pathophysiology

Tendons are hypocellular, collagenous connective tissues, which are integral to the function of the musculoskeletal system. Tendons connect bone to muscle and are essential for transmitting forces to produce joint movement; hence, tendon injury is a major cause of population morbidity. For example, in the USA there are more than fifty thousand rotator cuff tendon repairs performed annually [1]. Healthy tendon has great tensile strength due to the high proportion of type I collagen (>90% of total collagen) which is arranged in a hierarchical structure [2]. After injury, the thinner type III collagen (usually <1%) is found and has the property of rapidly forming crosslinks to stabilize the injury [3, 4]. Tendon tissues are poorly vascularized and predominantly utilize anaerobic energy systems resulting in poor healing potential after acute or overuse injury [5, 6]. Mesenchymal stem cells have been identified within tendons, but currently no candidate gene transcription factor, promoting differentiation towards a tendon lineage, has been isolated. Tendon progenitors and tenoblasts are immature, proliferative cells and are the precursors to the terminally differentiated tenocytes, which lay down collagen within the extracellular matrix (ECM). Tendon specific growth factors and cell markers are at this point unknown.

Following acute tendon injury, five overlapping healing phases have been identified, in a process that lasts up to 10 weeks in healthy tendons. However, the resulting tendon is thickened, fibrotic, and less resistant to tensile stress than the preinjury state. Surgical repair or scaffold reconstruction is considered if there is poor quality tissue present, or in situations where the normal healing processes cannot occur, such as unstable apposition of the free ends. In clinical situations where surgical repair is technically difficult, or has too many complications, including a significant re-injury rate, then scaffold reconstruction is the preferred surgical treatment. There are three sources for reconstructive graft, namely, autologous tendon (e.g., patella, hamstring, or palmaris longus), tendon allografts, or synthetic acellular engineered scaffolds. These reconstructive procedures have recognized complications including donor site morbidity in autografts, potential immune rejection and infection transmission in allografts, and possible delayed implant failure in synthetic grafts. Hence, in injury resulting in tendon discontinuity, there is a clinical need for improved tissue-engineered scaffolds [7–9].

**Figure 1:**
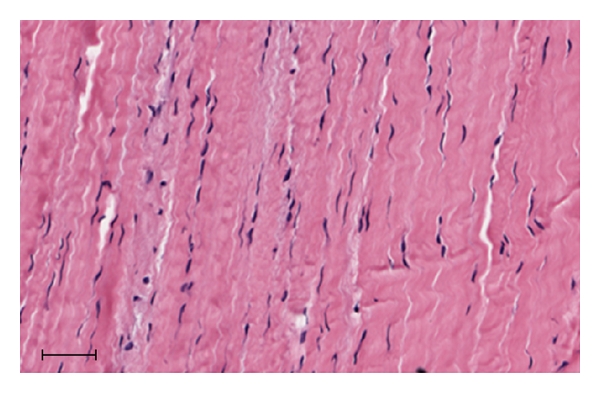
Normal Tendon. Note the relative paucity of cells.

**Figure 2:**
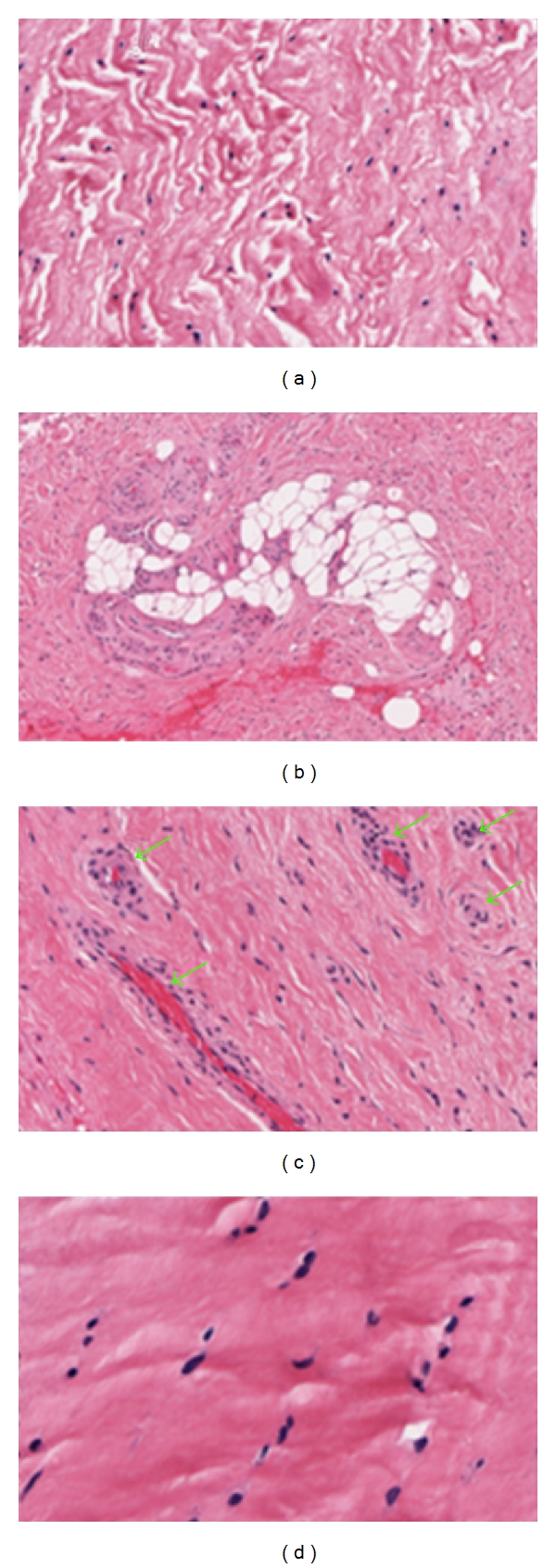
Tendinopathy in Rotator Cuff Tendons (a) Fibre disruption (b) Adipose tissue deposition (c) Vascular hyperplasia (d) Rounding of nuclei (Courtesy of University of Western Australia).

Tendons are also prone to overuse pathology which is associated with tenocyte depletion, microscopic collagen breakdown, and failed healing [10, 11]. This results in pain and altered function and contributes to tearing at lower strain thresholds. This process is defined as tendinopathy, which includes both tendinosis and tendinitis [12]. Tendinopathy demonstrates heterogeneous histological features with the presence of nontendon cell lines, such as fibroblasts, myofibroblasts, adipose, chondroid, and osteoid cells. There is an increase in ground substance with type III collagen (up to 30%), absence of inflammation, and marked reduction in the number of healthy tenocytes [10, 13]. Most tendons in the body can be affected, but the more disabling tendinopathies relate to the major joints such as the rotator cuff of the shoulder, the gluteal tendons of the hip (“greater trochanteric bursitis”), the common extensor tendons of the elbow (“tennis elbow”), and the Achilles tendon of the ankle. Initial treatment of tendinopathy is always conservative and is usually prolonged. There is reasonable evidence that exercise rehabilitation is beneficial, but limited evidence of efficacy for any of the other nonoperative treatments including platelet rich plasma injections which are purported to introduce autologous growth factors [14–16]. Surgical tendon debridement is sometimes undertaken for refractory cases, but this is expensive, disabling and the success is only modest [14]. Therefore, improved therapies for tendinopathy are required (Figures 1 and 2).

## 2. Cell Therapies

Preclinical studies have shown the potential for cellular therapies to increase tenocyte numbers and regenerate rather than repair tendon tissue. In the cellular treatment of tendon disorders, a small number of phase 1 and 2 clinical trials are being currently undertaken or have been completed. These trials have assessed the safety and efficacy of differing cell lines with varying degrees of cell potency, to treat tendinopathy.

### 2.1. Mesenchymal Stem Cells

Mesenchymal stem cells have the properties of proliferation and differentiation into mesenchymal tissue progenitors and are characterized by specific cell surface markers, adhesion molecules, growth factors, and ECM molecules [17]. MSCs can be isolated from a variety of tissues including; bone marrow, adipose tissue, the ACL, and tendon tissue [18–20].

MSCs can regenerate connective tissues, but there is increasing evidence that the mechanism of action may not be due to direct engraftment or differentiation [21]. MSCs secrete a variety of soluble autocrine and paracrine growth factors, which recruit MSCs, promote cell survival, and enhance the proliferation of endogenous connective tissue cells. These growth factors stimulate mitosis in tissue progenitors, induce angiogenesis, and reduce apoptosis [21–23].

MSCs are immune privileged which are thought to be due to their lack of MHC-II expression, disruption of T cell rejection mechanisms and secretion of anti-inflammatory mediators such as prostaglandins and interleukin-10 [24]. The use of allogeneic MSCs permits more efficient harvesting and expansion, but has the disadvantage of potential transmission of viral or prion vectors. Allogeneic MSCs can be used “off the shelf” in emergency situations, as they are always available (cryopreserved) and not rejected by host immune mechanisms. However, once differentiated, the evidence regarding persisting immune-privileged properties is inconclusive. MHC-II antigens can still be detected intracellularly by western blotting, even though they are not expressed on the cell surface [25]. Toma showed that a limited number of human MSCs persisted after differentiation into cardiomyocytes, after engraftment in a murine heart [26]. However, in contrast to Toma's findings, Huang demonstrated that in vitro differentiation of rat autologous and allogeneic MSCs, into myogenic lineages, reduced MHC I and increased MHC-II expression [27]. After 5 weeks only, autologous cells were present.

In preclinical animal models, both tendon laceration/defects and collagenase-induced tendinopathy are commonly studied. Chong et al. showed that intratendinous allogeneic MSCs, implanted in lacerated and sutured rabbit Achilles tendons, improved (accelerated) the histological and biomechanical parameters in the early stages of tendon healing [28]. In collagen gel scaffolds seeded in vitro and then implanted in rabbit tendons, ectopic calcification (due to osteogenesis) was found in up to 28% of cases, irrespective of the cell seeding density [29]. In a follow-up study, the authors noted that alkaline phosphatase activity was elevated around the sutures but only when the cells were in a 3D construct and not when in a monolayer [30]. The authors concluded that the osteoblastic proliferation was due to in vitro factors. Butler et al. advocated lower seeding density, with end posts rather than sutures and augmentation of the gel with type I collagen sponge and his group produced bioscaffolds with improved repair stiffness and improved force to failure [31]. No ectopic calcification was produced with this technique. Ouyang et al. demonstrated that PLGA scaffolds, seeded with allogeneic MSCs, repaired 1 cm defects in rabbit Achilles tendons with improved tensile stiffness compared to acellular scaffolds. However, the tendons with tissue engineered bioscaffolds only had 62% of the tensile stiffness compared to surgically repaired control tendons at 12 weeks [32].

In rabbit bone-patellar-bone ACL autografts, Soon et al. showed that autologous bone marrow (bm) MSCs improved osteointegration of the bone anchors compared to controls. However, Young's modules and graft stiffness were reduced [33]. Synovium-derived stem cells have also been shown to improve osteointegration in ACL tendon-bone healing [34, 35]. Ouyang et al. fabricated a bone marrow stromal cell sheet which was assembled on a poly l-lactide (PLLA) scaffold and produced an engineered ligament which was largely type I collagen [36]. The MSC that incorporated PLLA scaffold was stronger and more functional compared to acellular controls.

Current ACL reconstructive practice generally utilizes tendon auto or allografts that undergo a prolonged remodeling and revascularization process. Wei et al. transfected bmMSCs with an adenoviral vector expressing TGF-*β*1/VEGF165, which were then implanted into rabbit tendon ACL scaffolds [37]. The treated tendons demonstrated accelerated remodeling, angiogenesis, and improved mechanical properties compared to controls.

In an in vivo collagenase-induced tendinopathy study, Lacitignola et al. demonstrated that both autologous bmMSCs and bone marrow mononuclear cells (bmMNCs) could be injected intratendinously into equine tendons, and both produced effective tendon regeneration [38]. Similarly, Crovace et al. demonstrated that intralesional MSCs regenerated more type I collagen than control tendons, which had more type III collagen [39]. No calcification or ectopic tissue has been reported by serial ultrasound or at autopsy on these or a number of similar equine tendinopathy studies [30, 40].

MSCs are now used as a therapeutic intervention in the equine thoroughbred industry to treat flexor digitorum superficialis (FDS) tendinopathy. This injury is often career-ending and has of a recurrence rate of 56% with conventional treatments [41]. Pacini et al. treated 11 horses with FDS tendinopathy, with targeted intralesional injection of MSCs and 9 horses recovered [42]. Allogeneic equine adipose MSCs have been successfully used to treat 14 out of 16 horses with FDS tendinopathy without complication [43]. In a controlled trial Smith et al. demonstrated that intratendinous injection of 10 million autologous bmMSCs resulted in statistically significant improvements in tendon cross-sectional area, cellularity, crimp pattern, and DNA content compared to controls [44]. Currently, over 1800 thoroughbred horses have received therapeutic autologous MSCs for tendinopathy, and the recurrence rate is 27% (http://www.vetcell.com/), whereas the quoted recurrence rate with conventional, noncell-based treatment is 56% (*P* < 0.05). There have been no reported cases of ectopic tissue production detected on serial ultrasounds. Twelve horses have now undergone postmortems (17 tendons), which have revealed good healing with minimal inflammatory cells, with crimped organized collagen fibers and no ectopic or neoplastic tissues [45].

**Figure 3:**
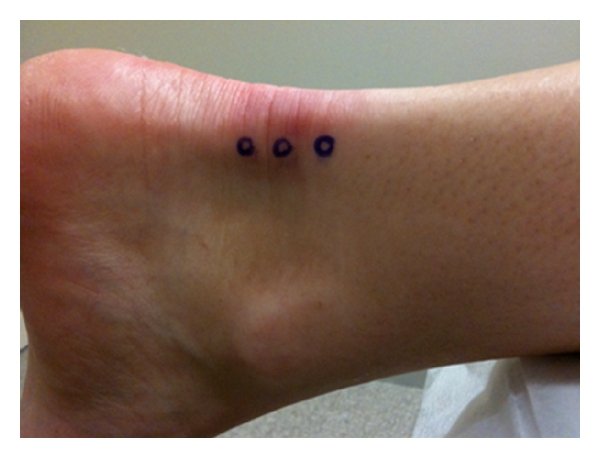
Injection sites for Achilles tendon.

**Figure 4:**
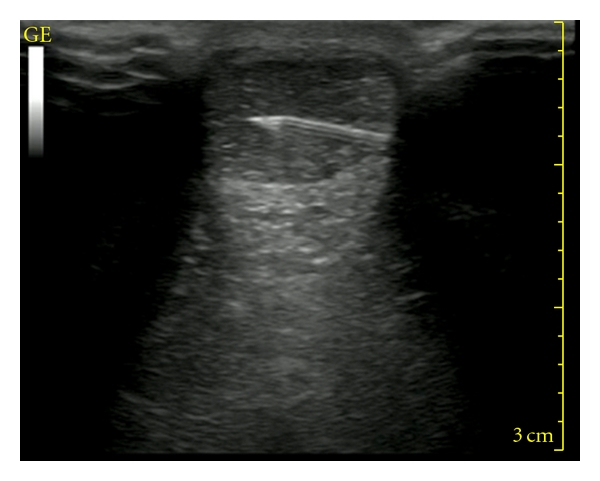
Ultrasonographically guided intratendinous Achilles injection.

The author of this review paper is currently undertaking a phase 1 trial in the use of allogeneic mesenchymal stromal cells in the treatment of human chronic (refractory) Achilles tendinopathy (Figures 3 and 4).

### 2.2. Embryonic Stem Cells

Embryonic stem cells (ESCs) are pluripotent cells with greater plasticity and proliferative capacity compared to adult MSCs. This means that they can provide an unlimited supply of MSCs and connective tissue progenitors. MSCs do improve tendon architecture, but have not induced the degree of tendon regeneration that is seen in injured fetal tendon [46]. A disadvantage of ESCs is their capacity to form teratomas. Chen et al. were the first to show that tendon regeneration could be achieved by in vitro differentiation into MSCs and then tenocytes [47]. These researchers used a xenograft model with human ESCs, which were differentiated into MSCs, then seeded in a fibrin scaffold before being implanted into a rat patellar tendon model. The hESC-MSCs had much better structural and mechanical properties than did the controls. The hESC-MSCs remained viable at the tendon wound site for at least four weeks and secreted human fetal tendon-specific matrix components and differentiation factors, which then activated the endogenous regeneration process in tendons. No ectopic tissue or teratomas were reported in this study, but the authors state that calcification was noted (unreported) in some of their other cases of patellar tendon fibrin ESC scaffolds. The authors concluded that improved differentiation techniques were required for ESCs for use in bioscaffolds for tendon repair. In a blinded placebo-controlled randomized trial of ESCs in equine collagenase-induced tendinopathy, intratendon injections of undifferentiated ESCs were shown to improve tissue architecture, tendon size, and tendon linear fiber pattern [48]. The eight horses were followed up with ultrasound and MRI scans, and no calcification or teratoma production was noted.

Induced pluripotent stem cells (iPS cells) are formed by reprogramming a nonpluripotent somatic cell, such as dermal fibroblasts using transfection of stem cell genes such as, c-myc, sox-2, oct-4, and klf-4. This avoids the ethical issues relating to embryonic stem cells but currently there are no clinical trials using iPS cells or ESCs in tendon research.

### 2.3. Tendon-Derived Cells

Until recently, little was known about the characteristics of tendon cells and their precursors. In 2007, Bi et al. isolated a rare cell population from an ECM niche and demonstrated clonogenicity, self-renewal, and multipotent differentiation capacity [20]. The cells in the population showed heterogeneity in these properties and so were referred to as tendon stem/progenitor cells (TSPCs). These cells reside in a niche, which includes biglycan (Bgn) and fibromodulin (Fmod), which in turn controls the fate of TSPCs by modulating BMP activity. Lower levels of Bgn and Fmod in the ECM are associated with osteogenesis, which can be found in tendinopathy [20]. No tendon-specific marker was identified in TSPCs, but compared to bone marrow MSCs, they highly expressed the tendon-related factors Scx, COMP, and Tenascin-C. When injected into mice, TSPCs were more likely to form tendon than bmMSCs, which preferentially formed bone. Tempfer et al. biopsied human rotator cuff tendons and isolated cells expressing both scleraxis and CD133, which is a marker of endothelial and hematopoietic stem cells [49]. The authors suggested that these were perivascular tendon cells in a vascular tendon niche, which had been activated for tendon repair. Further characterization of these cells is required to establish stem cell characteristics. Currently, there are no published trials on the use of tendon-derived stem/progenitor cells (TSPCs) in tendon engineering.

**Figure 5:**
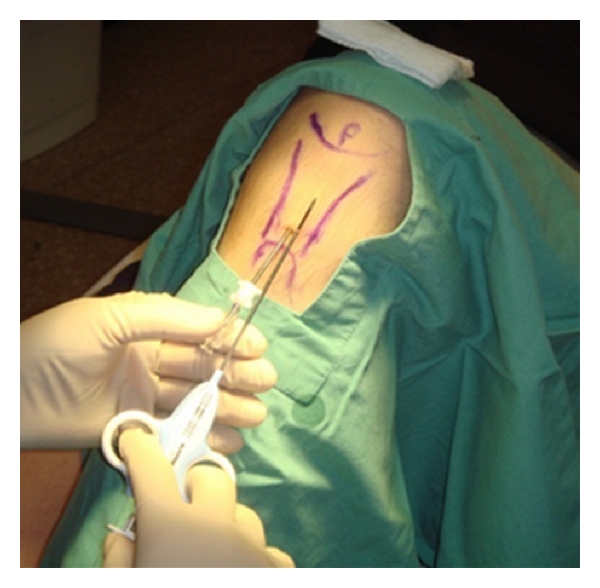
Trucut biopsy of patella tendon for tenocyte harvesting (Courtesy of M Zheng).

Autologous tenocytes can be harvested and expanded, prior to reimplantation. In 2002, Cao et al. seeded PLGA scaffolds with autologous tenocytes and successfully repaired hen flexor digitorum profundus tendon defects [50]. The cell-seeded scaffolds had aligned collagen, which had 83% of normal tendon strength, whereas the unseeded scaffolds only had 9% of normal strength. Similarly, autologous tenocytes were seeded on both porcine small intestine submucosa and type I/III collage bioscaffold (ACI-Maix), to repair rabbit rotator cuff models [51]. In a randomized controlled trial of rabbit collagenase tendinopathy model, Chen et al. showed that autologous tenocytes (either from tendon or epitendineum tissue) improved tendon remodeling, histological outcomes, collagen content, and tensile strength [52]. The autologous tenocytes that improved type I collagen expression, did not affect type III collagen and secreted protein rich in cysteine (SPARC) expression. In a phase I/II clinical trial of expanded autologous tenocytes, in 25 subjects with lateral epicondylitis [53], demonstrated improved grip strength, reduced pain, and a reduced Quick DASH Score over a six-month followup (*in press*). No ectopic tissue, tumors, infection, or other complications were reported. Autologous tenocyte implantation (ATI) is currently available in Australia, and over 100 procedures have been undertaken with no reported complication (*personal communication*). Tendon harvesting is usually performed with a Trucut needle, and the donor site is typically the patellar tendon, and ultrasound guidance is recommended. In athletes who play weigh-bearing sports, a miniopen biopsy of the palmaris longus tendon of the wrist is the preferred tendon donor site. These biopsies do require technical skill and are mildly invasive. Currently, in the Netherlands, a registered double-blind randomized controlled trial is being undertaken to assess the efficacy of ATI in 90 subjects with Achilles tendinopathy (Figure 5).

### 2.4. Dermal Fibroblasts

Dermal fibroblasts (DFbs) have been used in tissue engineering due to their abundant supply, ease of harvesting, and reprogrammability. They have multi-differentiation potential and have been shown to develop into brain, glia, muscle, and adipose lineages [17]. In vitro experiments have shown promise in tendon engineering [54, 55]. A concern regarding the use of DFbs in tendon engineering is the production of scar tissue. In a controlled trial, Deng et al. showed the importance of applying static mechanical strain on PGA constructs seeded with dermal fibroblasts (DFbs). After 14 weeks, histology revealed longitudinally arranged collagen with spindle-shaped cells in the strain group, compared to disordered fibrous tissue with randomly aligned collagen and reduced strength to failure in the controls (no strain) [56]. The researchers also compared the histology to PGA scaffolds seeded with tenocytes and reported no difference between the cell source [54]. When static tension is applied to DFbs in bioreactors, the cells produce type I and type III collagen, but other similarities to tenocytes cannot be confirmed, as there are no tenocyte-specific markers.

Connell et al. demonstrated that DFbs could be expanded, stretched, and induced to lay down collagen in a similar fashion to tenocytes [57]. In a prospective study on twelve subjects with refractory lateral epicondylitis (“tennis elbow”), these researchers implanted 10 × 10^6^ DFbs with precision-guided ultrasound intratendinous injection. Over the 6-month follow-up, there was improvement in disability scores and ultrasound tendon parameters (*P* > 0.05) in all but one subject. However, the collagen-producing fibroblasts were administered with platelet-rich plasma, which has been reported to improve healing in clinical trials [58, 59]. In a randomized trial of 60 cases of patellar tendinopathy, comparing ultrasound guided intratendinous injection of dermal fibroblasts to plasma controls, a faster response to treatment and significantly greater reduction in pain and improved function was noted in the treatment group [60]. One patient in the treatment group experienced tendon rupture, and subsequent biopsy showed relatively normal tendon tissue with type I collagen and tenocytes with normal morphology, and no ectopic tissue was noted.

**Figure 6:**
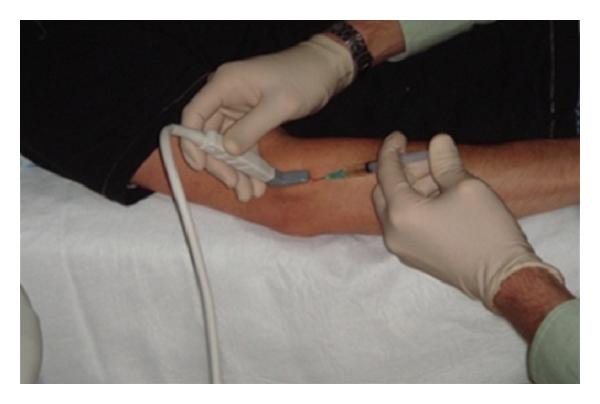
Injection of lateral epicondyle under ultrasound guidance.

Currently there are no current registered trials in the use of dermal fibroblasts in tendon-ligament engineering (Figure 6).

## 3. Gene Therapy

The therapeutic plasticity of stem cells means that specific transcription factors can be introduced which leads to reprogramming and phenotype transition [61]. A master transcription factor for the tendon lineages is yet to be discovered. Scleraxis (Scx) is the most studied potential marker of neotendon formation discovered to date [18]. However, other candidate genes include SIS1, SIX2, EYA1, EYA2, THBS4, and TNMD [62, 63], showed that MSC differentiation into neotendon was mediated by smad8 expression, which the authors felt was inhibitory of the normal osteogenesis pathway induced by bone morphogenic protein 2 (BMP2) [63].

Stem cells can also be gene modified to secrete growth factors which have autocrine and paracrine effects and which can lead to mesenchymal stem cell (MSC) recruitment, MSC differentiation into tenocyte lineages, and collagen synthesis. However, it appears that no specific tenogenic growth factor has yet been identified. A number of proteins have been shown to induce neotendon formation including fibroblast growth factor (FGF)-2, transforming growth factor (TGF)-*β*, insulin-like growth factor (IGF)-1, vascular endothelial growth factor (VEGF), platelet-derived growth factor (PDGF), and members of the BMP superfamily (such as the growth and differentiation factors—GDFs) [40, 64–67]. The local administration of VEGF improves tendon revascularization, but not graft mechanics [34, 68]. TGF-*β*1 promotes improved strength in Achilles tendon regeneration by regulating collagen I and III synthesis, cross-link formation, and matrix remodeling [69].

As most growth factors have a restricted biological half-life, slow release preparations or transient secretion by MSCs is required during healing and regeneration processes. A number of animal studies have confirmed that transitory expression of growth factors including TGF-*β*1, GDF5, and IgF-1 produce some improvement in tendon histology, biomechanics, or healing rate [69–71]. However, most gene delivery methods require viral vectors with associated potential risks including immune rejection, uncontrolled transgene expression, and insertional mutagenesis [24]. At present, there are no registered clinical trials using gene-modified cell therapies in tendon disorder.

## 4. Bioscaffolds

Currently, autologous tendon or tendon allografts are the preferred scaffolds of choice for tendon defects requiring surgical reconstruction or augmentation. Because tendons have similar histological and physical properties to ligaments (which connect bones to bones), there is considerable overlap and interchange in scaffolding technology between these two structures. In clinical practice, tendon auto or allografts are the preferred donor tissue of choice for ligament repair, primarily because tendons are larger and more easily accessible and can be sacrificed with less morbidity than ligament donor sites.

Clinical examples requiring consideration of surgical scaffolding include

- an elderly patient with a painful chronic degenerate massive rotator cuff tendon tear, who has poor quality tissue (further retracted by unopposed muscular contraction) with a high chance of failure of primary surgical repair.
- a young professional athlete, with a high-grade anterior cruciate ligament (ACL) injury, who may only experience mild painless knee instability when pivoting at speed, but the injury is effectively career-ending.

Potentially synthetic scaffolds offer clinical and cost benefits over current grafting techniques, due to accelerated healing with no harvesting morbidity, resulting in shorter hospitalization and rehabilitation periods. Tissue-engineered scaffold materials suitable for cell seeding (“bioscaffolds”) are classified as natural or synthetic. Natural bioscaffolds include collagens, small intestine submucosa, and silk fibers, whereas most of the synthetic bioscaffolds have been derived from poly-l-lactic acid (PLA) and poly-lactic-coglycolic acid (PLGA) [72, 73]. Important factors for bioscaffold design include biocompatibility, biodegradation rates, mechanical properties, porosity for cell infiltration, nutrient transmission, and the biologic role of the ECM [18]. Type I collagen gels have been the most studied type of bioscaffold. The seeding density affects mechanical stability and the cellular alignment and reorganization of the matrix [74]. Collagen gels have been enhanced by in vitro seeding and collagen hybridization with PLA or cross-linking with dicatechol nordihydroguaiaretic acid (NDGA) [75]. At present, no tenocyte-collagen scaffold constructs have been able to achieve similar mechanical properties to native tendon [74].

An ACL scaffold must biomechanically match the native ligament, and the graft must allow for osseous attachment with current surgical techniques. Cartmell and Dunn produced a potential ACL scaffold in vitro by decellularizing a patellar tendon allograft to reduce antigenicity and then seeded the graft with fibroblasts [76]. These modified allografts have the potential to be developed into mechanically functional delivery vehicles for cells, gene therapy vectors, or other biological agents. Silk is emerging as a promising material for connective tissue scaffolds. Sahoo and colleagues developed a biohybrid scaffold system by coating bioactive basic (b) FGF-releasing ultrafine PLGA fibers over mechanically robust slowly degrading degummed knitted microfibrous silk scaffolds [77]. The bFGF stimulated MSC proliferation and tenogenic differentiation, and the resulting collagen production contributed to the enhanced mechanical properties of the tendon analogue.

Currently, there are no registered clinical trials using cell-seeded scaffolds to repair tendons (or ligaments).

## 5. Mechanostimulation

In clinical practice, exercise rehabilitation is a reasonably efficacious intervention for the treatment of tendinopathy; however, the exact exercise prescription (frequency, amplitude, and intensity) and type of exercise (eccentric, concentric or stretching) are still not fully established [78]. Mechanical loading of tendons is known to produce a trophic cellular response with stem cell proliferation and differentiation into tendon progenitors, with a resulting increase in deposition of extracellular matrix [20, 79, 80]. There is an associated increased scleraxis upregulation and secretion of cellular cytokines, including TGF-*β* and IGF-I [18, 81, 82].

In vitro, the type and axis of loading of bioscaffolds affect the cellular response. Compression loading leads to the formation of more cartilaginous tissue, whereas shear stress produces increased matrix metalloproteinases (MMP-1 and 3) in rabbit tendon fibroblasts, which results in matrix disruption [83, 84]. Repetitive loading, at higher construct strains, results in production of PGE_2_ and BMP2, leading to differentiation into nontendon lineages [85, 86]. Zhang and Wang demonstrated that in vitro uniaxial loading of rabbit tendons at 0.5 Hz for 12 hours upregulated tenogenesis and type I collagen production at 4% strain, but increased adipogenesis and osteogenesis at 8% strain.

Repetitive uniaxial mechanical stretching of seeded bioscaffolds increases ECM production and fibrillar alignment in a number of cell lines, including cultured tendon fibroblasts, isolated tendon fascicles, dermal fibroblasts, and MSCs. Chen et al. found that poorer outcomes resulted when stress was applied in the first three days after cell seeding silk fibroin matrices [87]. The authors concluded that prerequisites include both established cell-to-cell contact and sufficient ECM before stress is applied.

The optimal mechanical stimulation regimes for tendon bioscaffolds are yet to be established, but some studies have demonstrated that loading results in a sixfold increased failure stress [88]. Future cellular bioscaffold design will require a multidisciplinary strategy combining cell technology, engineered scaffolds, and mechanical stimulation [31].

## 6. Summary

Current clinical treatments for tendon defects and chronic tendinopathy are only moderately effective. Tendons are poorly vascularized, relatively acellular, and have limited regenerative potential. Tendon healing is prolonged and results in biomechanically inferior scar tissue that is prone to reinjury. Surgical reconstruction or augmentation with current scaffolds is often associated with donor site morbidity and usually requires lengthy and costly postoperative rehabilitation. New therapies are required, and cell-based treatments offer great potential due to their ability to regenerate connective tissues, the improved understanding of the properties required for cell-seeded bioscaffolds, and the ease of precision implantation with minimally invasive percutaneous guided injection. Current studies range from pluripotent cells to fully differentiated tenocytes, but are yet to determine the ideal cell type for therapeutic tenogenesis. Cell lines such as ESCs have greater potency and proliferative properties, but also have the potential for more complications including tumorogenesis. MSCs offer some promise in tendon engineering due to their proliferative capacity and the potential of genetic modification to secrete tenogenic growth factors. MSCs are also immunosuppressive and are allogeneic, obviating the need for host biopsy if nontissue-matched cells are used. The ideal cell source for MSC harvesting for use in tenogenesis is yet to be determined. Ectopic bone formation has been reported in MSC-seeded tissue-engineered tendon bioscaffolds, but this complication appears to be due to in vitro factors [31]. Unintended differentiation has not been reported with intratendinous injection in large animal tendinopathy studies or with therapeutic use in thoroughbred horses. For the repair of tendon (and ligament) defects, the ideal tissue engineered bioscaffold, seeding density and preferred mode of mechanical stimulation for both in vivo and in vitro seeding are unknown.

Dermal fibroblasts are a nonhomologous cell, which have the advantage of easy harvesting with a minimally invasive biopsy. However, the lack of tenocyte markers and histological confirmation of current studies makes it difficult to determine whether the microscopic similarities between fibroblasts and tenoblasts result in true tenogenesis. The harvesting procedure for autologous tenocytes (ATI) is more invasive, and tenocytes have limited proliferation potential, but there is no risk of unintended differentiation. Even though the safety of ATI appears established, the results of randomized controlled trials to determine efficacy are awaited. In a study comparing tenocytes, tendon sheath fibroblasts, adipose tissue-derived MSCs, and bmMSCs in healing rabbit flexor tendon defects, little difference was noted in the ability to reseed a decellularized tendon scaffold [89]. However, several other studies have suggested that tendon sheath fibroblasts (tenoblasts) possess a greater rate of proliferation than tenocytes [49, 50].

There is a great deal yet to be discovered in our understanding of the role that cellular therapies will play in the treatment of tendon disorders, and at present there is insufficient data to conclusively prove that these treatments are safe and efficacious. However, this technology appears to hold great promise and will probably become an important clinical therapy in the near future in orthopedic, sports, and musculoskeletal medicine.
